# Surface Glycosylation Profiles of Urine Extracellular Vesicles

**DOI:** 10.1371/journal.pone.0074801

**Published:** 2013-09-19

**Authors:** Jared Q. Gerlach, Anja Krüger, Susan Gallogly, Shirley A. Hanley, Marie C. Hogan, Christopher J. Ward, Lokesh Joshi, Matthew D. Griffin

**Affiliations:** 1 Regenerative Medicine Institute (REMEDI), National Centre for Biomedical Engineering Science, National University of Ireland, Galway, Ireland; 2 Glycoscience Group, National Centre for Biomedical Engineering Science, National University of Ireland, Galway, Ireland; 3 Dept. of Medicine, Division of Nephrology and Hypertension, Mayo Clinic, Rochester, Minnesota, United States of America; Oxford University, United Kingdom

## Abstract

Urinary extracellular vesicles (uEVs) are released by cells throughout the nephron and contain biomolecules from their cells of origin. Although uEV-associated proteins and RNA have been studied in detail, little information exists regarding uEV glycosylation characteristics. Surface glycosylation profiling by flow cytometry and lectin microarray was applied to uEVs enriched from urine of healthy adults by ultracentrifugation and centrifugal filtration. The carbohydrate specificity of lectin microarray profiles was confirmed by competitive sugar inhibition and carbohydrate-specific enzyme hydrolysis. Glycosylation profiles of uEVs and purified Tamm Horsfall protein were compared. In both flow cytometry and lectin microarray assays, uEVs demonstrated surface binding, at low to moderate intensities, of a broad range of lectins whether prepared by ultracentrifugation or centrifugal filtration. In general, ultracentrifugation-prepared uEVs demonstrated higher lectin binding intensities than centrifugal filtration-prepared uEVs consistent with lesser amounts of co-purified non-vesicular proteins. The surface glycosylation profiles of uEVs showed little inter-individual variation and were distinct from those of Tamm Horsfall protein, which bound a limited number of lectins. In a pilot study, lectin microarray was used to compare uEVs from individuals with autosomal dominant polycystic kidney disease to those of age-matched controls. The lectin microarray profiles of polycystic kidney disease and healthy uEVs showed differences in binding intensity of 6/43 lectins. Our results reveal a complex surface glycosylation profile of uEVs that is accessible to lectin-based analysis following multiple uEV enrichment techniques, is distinct from co-purified Tamm Horsfall protein and may demonstrate disease-specific modifications.

## Introduction

Chronic kidney disease (CKD) is a growing public health issue worldwide [1]–[3]. Percutaneous kidney biopsy is currently the definitive diagnostic method for determining CKD etiology. Although the occurrence of complications is relatively low, the invasive nature of kidney biopsy has inherent risks which may rule out the use of the procedure with some patients such as those with compounding medical conditions [4]–[6]. Cost and access to care are also considerations for the use of renal biopsy [4]. Therefore, the discovery of non-invasive alternatives to biopsy for diagnosing and monitoring CKD is highly desirable.

The nephron, and its active filtration mechanism within the glomerulus, facilitates the transfer of waste to urine at the interface of the circulatory and renal systems. Populations of urinary extracellular vesicles (uEVs), which include vesicles of 20–100 nm typically referred to as “exosomes” along with other vesicle subtypes, are actively released by epithelial cells throughout the nephron and have been shown to contain a wide variety of surface and intracellular proteins as well as nucleic acids which may include important biomarkers [7]–[16]. A limited number of studies have documented specific alterations to the protein composition of uEVs in the context of acute as well as chronic kidney diseases in small animal models and human subjects, supporting the contention that uEV-based assays will be of clinical value for diagnostic and prognostic purposes [17], [18]. Notably, the uEV proteome includes many proteins that are known to be post-translationally modified through the attachment of carbohydrate moieties (glycosylation) and to localize to the plasma membrane [16], [19], [20].

As the pathways of protein translation, folding, sorting and secretion are directly related to those of protein glycosylation [21], it is reasonable to assume that oligosaccharide components and overall expression of glycoproteins will be altered in kidney conditions associated with cellular stress or altered metabolic activity [22]–[25]. In support of this, alterations to carbohydrate structures have been identified in association with renal development, kidney disease and kidney transplantation [22]–[27]. Abnormalities of *N*-linked oligosaccharides have been associated with kidney inflammation in a mouse model of systemic lupus erythematosis [27] and the potential for such abnormalities to be detectable in uEVs has been demonstrated by Staubach *et al*. who observed a shift in abundance from high-mannose to complex-type *N*-glycans on uEVs from individuals with classical galactosemia [28]. Based on these studies, it is highly likely that proteins populating the surface of uEVs reflect glycosylation of renal epithelial cells and that alterations to uEV surface glycosylation may be directly reflective of pathogenic events. However, in comparison to the analysis of EV proteins and nucleic acids from urine and other biological fluids, interrogation of the glycosylation characteristics of EVs has been pursued only to a minimal degree [20], [28]–[30].

Precise structural analysis of cell and protein-bound carbohydrates remains a technically challenging field and strategies which employ affinity agents represent a valuable alternative approach for rapidly profiling glycosylation [31], [32]. Lectins are non-antibody, carbohydrate-binding proteins which have affinity for particular carbohydrate components commonly found on the surface of mammalian cells and vesicles derived from them [33]. Microarrays of lectins printed on functionalized glass slides have recently emerged as one of the most promising technologies for rapid profiling of glycoconjugates [29], [34]–[40]. Lectin microarray technology has the potential for direct translation to clinical use or may serve as a screening tool for the development of customized affinity-based assays. As a proof of concept, glycoprofiling of vesicles [29], [30] and the abundant urinary glycoprotein uromodulin (or Tamm-Horsfall protein, (THP)) [39] have been independently demonstrated. However, detailed profiling of vesicle glycosylation has not yet been carried out.

The most commonly employed uEV isolation techniques are ultracentrifugation (UC) and density-gradient ultracentrifugation (gUC) [19], [41]–[46]. However, large-scale uEV isolation by these methods is time-consuming and labor intensive. In contrast, the centrifugal filter concentration (or spin concentrator, SC) method offers the potential for faster sample processing without the need for highly-specialized equipment. Filtration methods have already been reported as alternatives for vesicle isolation, but these studies have indicated that vesicle populations and protein content may differ between UC-, gUC- and SC-enriched samples [46]–[48]. Thus, for new analytical approaches, including the profiling of uEV glycosylation characteristics, it is important to compare results for uEVs enriched by UC, gUC and SC methods.

In this study, we hypothesized that the surface glycosylation profile of uEVs is readily distinguishable from that of the frequently co-purified urinary glycoprotein THP and can be determined by lectin screening. To explore this, we adapted flow cytometry (FCM) and lectin microarray strategies to profile surface glycosylation characteristics of uEVs isolated by three methodologies from healthy subjects in addition to purified THP. Our results indicate that uEVs isolated by UC, SC and gUC share a similar profile which is clearly distinct from that of THP alone. Furthermore, a pilot study comparing lectin microarray profiles of uEVs from autosomal dominant polycystic kidney disease (ADPKD) patients and age-matched controls demonstrated the potential clinical value of this technique.

## Results

### Urine Extracellular Vesicle Enrichment and Tamm-Horsfall Protein Purification

**Figure 1:**
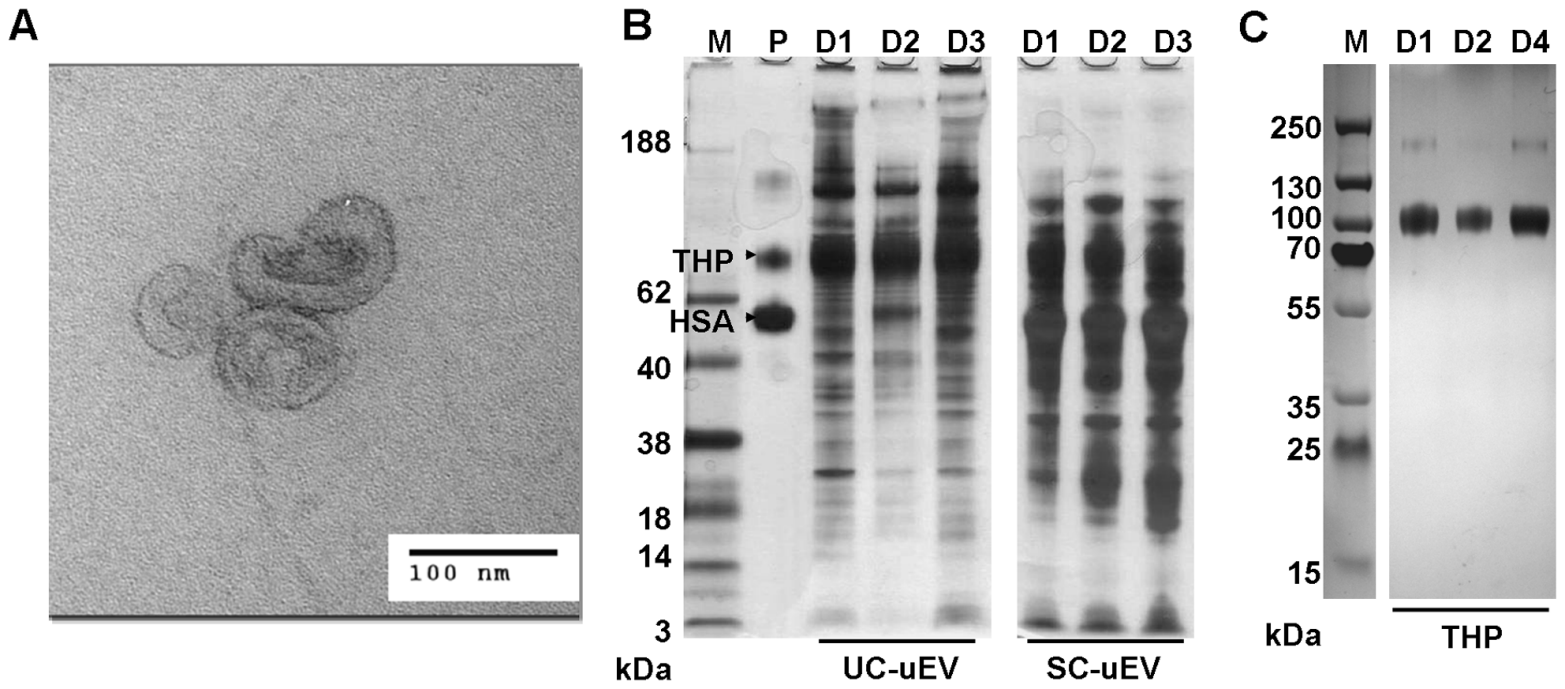
Evaluation of uEVs enriched by UC and SC methods. (**A**) TEM image of representative UC-uEVs. (**B**) Migration of 1 µg THP and 1 µg human serum albumin (HSA, lane P) compared to 5 µg total UC- and SC-uEV protein profiles from 3 healthy donors (lanes D1, D2 and D3). (**C**) 4–20% Bis-Tris SDS-PAGE of 2 µg THP isolated from three healthy donors (lanes D1, D2, and D4). Lane M represents M_r_ standard and gels were stained with silver. Data is representative of outcomes from a minimum of 10 individual uEV enrichment experiments for each method.

Microvesicles were enriched from paired aliquots of first morning urine samples of healthy adults by UC and SC methods and normalized based on protein estimation. For samples prepared by both methods, transmission electron microscopy (TEM) revealed vesicles of the expected size and morphology (see **Figure 1A** for example). Total uEV protein was examined by SDS-PAGE (**Figure 1B**). As reported by others, THP co-purification was also observed to variable degrees for both methods. Striking differences were seen between UC-uEV and SC-uEV high molecular weight protein quantity and general protein distribution (**Figure 1B**). Notably, SC-uEVs contained prominent bands of equal M_r_ to human serum albumin (HSA) and THP, while UC-uEVs contained relatively less HSA. To allow for comparative analyses of purified THP with uEV samples, THP glycoprotein was separately isolated to high purity from urine of multiple healthy donors (**Figure 1C**). Paired UC and SC samples from a single healthy donor were also subjected to particle count and size analysis [49]. As shown in **Figure S1 in File S1**, the size distributions of uEVs prepared by UC-uEV and SC-uEV methods from the same urine specimen were virtually identical and were consistent with previous reports [19], [50].

**Figure 2:**
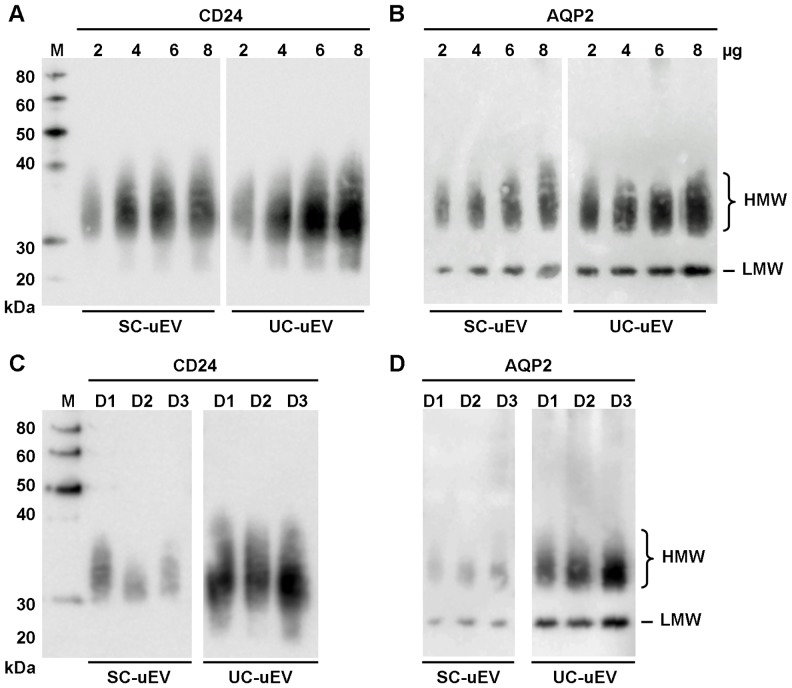
Immunoblots of SDS-PAGE-separated UC- and SC-uEV proteins. Stepwise responses of 2 to 8 µg uEV protein with antibodies specific for (**A**) CD24 and (**B**) AQP2. Inter-individual variability of (**C**) CD24 and (**D**) AQP2 content for equal amounts of SC- and UV-uEV proteins from three individuals (lanes D1, D2 and D3). 5 µg/well loaded for (C) CD24 detection, 4 µg/well loaded for (D) AQP2 detection. Lane M represents M_r_ standard. Data is representative of a minimum of 10 individual uEV enrichment experiments for each method.

Investigation of both UC- and SC-uEV proteins with antibodies for EV-specific proteins CD24 [46] and AQP2 [11] revealed more prominent staining for UC-uEVs relative to amount of protein loaded (**Figure 2A and 2B**). Inter-individual variability for CD24 and AQP2 was low as shown in **Figures 2C and 2D**. Overall, these analyses indicated that similar vesicle populations are isolated from urine of healthy adults by both UC and SC methods but that co-purified proteins, including albumin, are more abundant in SC-uEV samples resulting in overestimation of actual vesicle content and, potentially, obscuring analyses of uEV-specific biomolecules.

### Flow Cytometry with uEVs and THP

**Figure 3:**
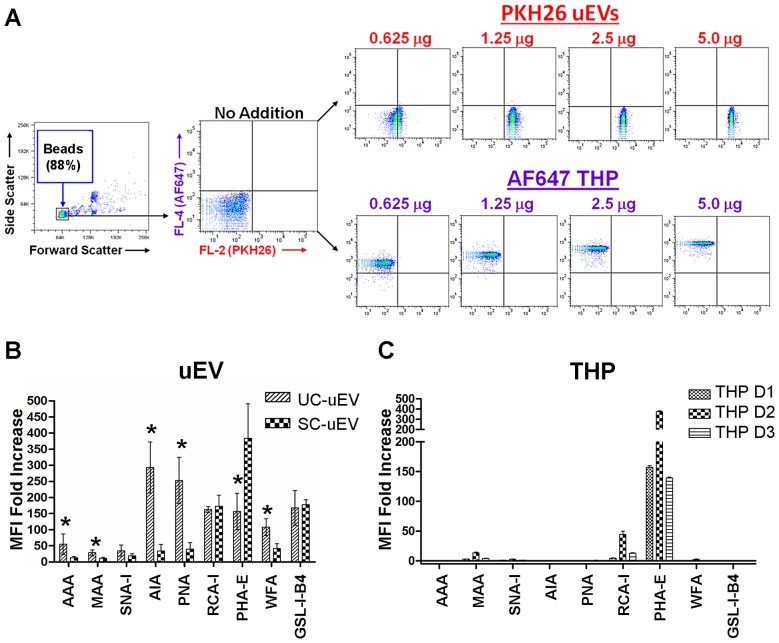
Quantitative analysis of lectin binding to bead-bound uEVs and THP by flow cytometry. (**A**) Verification of PKH26 labeled uEVs and AlexaFluor® 647 labeled THP conjugation to latex beads. Relative mean fold intensity of staining for 9 lectins with (**B**) 5 µg unlabeled UC- and SC-uEVs and (**C**) 20 µg unlabeled THP from 3 healthy donors (D1, D2 and D3) conjugated to latex beads. Data is representative of 3 biological replicates. Error bars represent ±1 standard deviation. Significance (* = *p*<0.05 for UC-uEV vs. SC-uEV) was determined by paired, two-tailed Student’s t-tests.

The feasibility of using lectins to screen for uEV surface carbohydrate signatures was explored by adaptation of a previously reported FCM approach [44]. Initially, binding of fluorescently-labeled uEVs, as well as labeled THP, to latex beads was confirmed *via* FCM (**Figure 3A**). Subsequently, uncoated beads and beads coated with unlabelled uEVs (prepared by UC and SC methods) were incubated with nine biotinylated lectins followed by fluorochrome-labeled streptavidin (SA). As shown in **Figure 3B**, binding of the lectins MAA, PNA, Jacalin (AIA), GSL-I-B4, PHA-E, RCA-I, SNA-I and WFA was observed at varying intensities for paired UC- and SC-uEV samples from multiple healthy adults. Additional plots and gating details can be seen in **Figure S2 contained in File S1**. For 6 of 9 lectins tested, binding intensity was greater for UC-uEVs compared to SC-uEVs, consistent with greater vesicle density per µg of protein. However, binding intensity of GSL-I-B4 and RCA-I was equivalent for UC-uEVs and SC-uEVs, and binding intensity of PHA-E was greater for SC-uEVs. FCM of THP-coated beads with the same lectins demonstrated robust binding of PHA-E, relatively low binding of MAA and RCA-I and no detectable binding of the remaining six lectins (**Figure 3C**). These results confirmed that lectins can be utilized as detection agents for surface carbohydrates on uEVs purified by multiple methods and also provided evidence that the lectin-binding profile of uEVs has features distinct from that of THP.

### Comparison of uEVs and THP by Lectin Microarray Profiling

**Figure 4:**
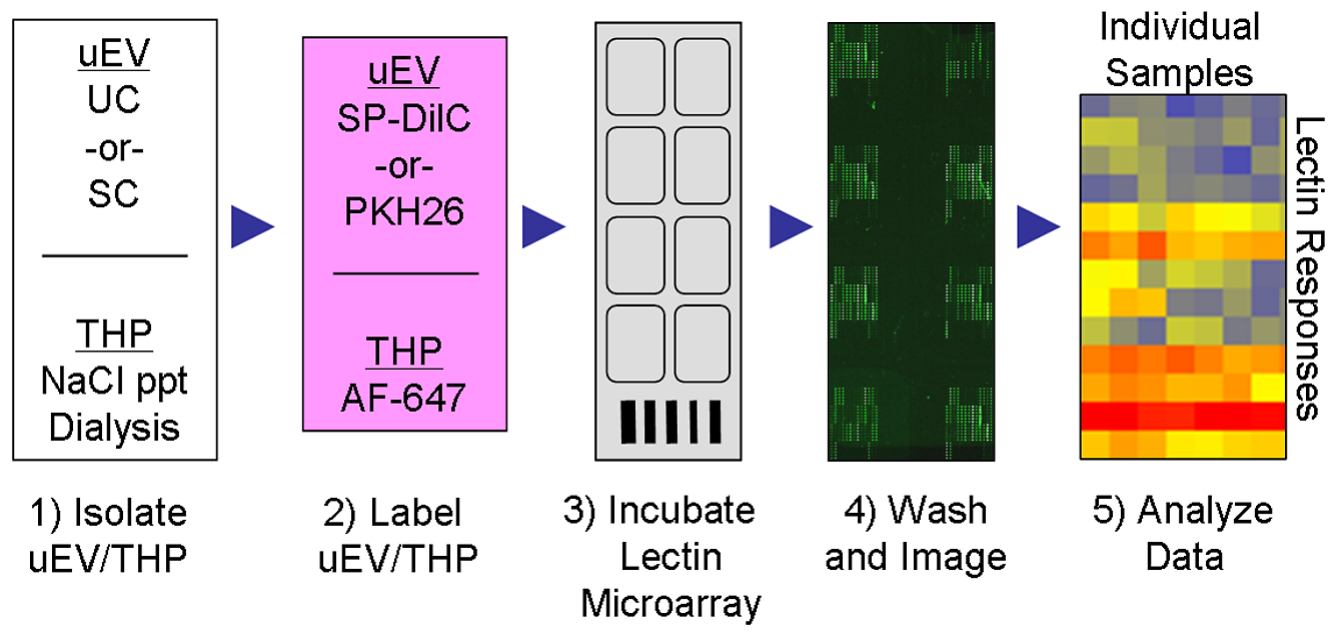
Schematic of lectin microarray profiling process.

To broaden the lectin-based glyco-profiling analysis and to further evaluate the differences between UC- and SC-uEVs, a lectin microarray approach was developed and optimized. As summarized in **Figure 4**, labeled and washed uEVs and THP were incubated with lectin microarrays printed and tested in-house. Optimal dilution of uEVs labeled with either PKH26 or SP-DilC18(3) ranged from approximately 0.2 to 0.5 µg/mL based on Bradford estimation of protein content. This range gave the greatest signal-to-noise advantage and minimized spurious artifacts likely caused by the aggregation of vesicles during incubation with the lectin microarrays. For THP, 0.5 µg/mL produced intensities detectable at a broad range of lectins without saturation at highest binding lectins. Triple technical replicates demonstrated lectin-binding signals ranging to approximately 10,000 RFU for the uEV samples indicating a substantial dynamic range.

**Figure 5:**
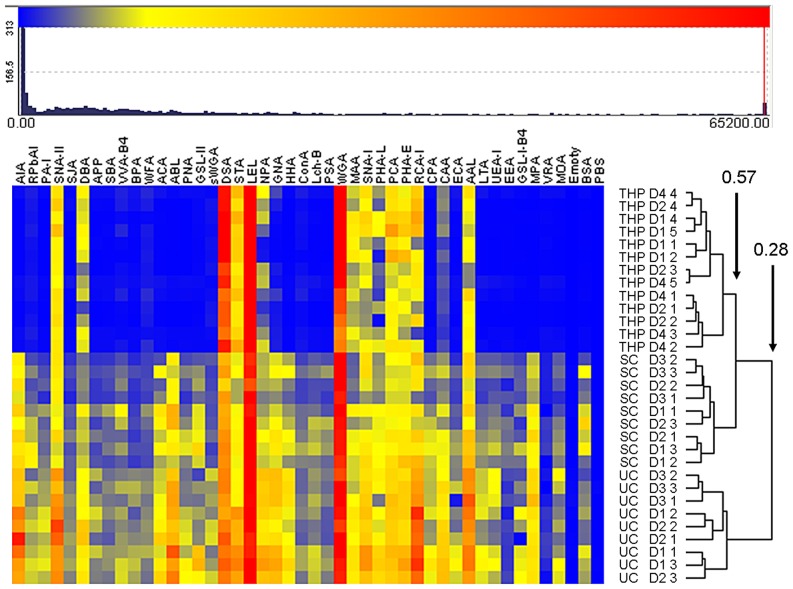
Unsupervised hierarchical clustering of lectin microarray profiles for intact urine uEVs and purified THP from three healthy individuals. LM data for all uEV (donors D1, D2, and D3) and THP samples (donors D1, D2 and D4) normalized by rescaling (range 0 to 65200). Individual technical replicates are indicated by designation of 1 to 5. Two groups are evident at 0.28 similarity, three groups at 0.57 similarity. Clustering based on Euclidean distance, complete linkage method. Responses and clustering shown are typical of those obtained from a minimum of 10 healthy uEV enrichment and profiling experiments.

Unsupervised hierarchical clustering of normalized profile data provided clear evidence that THP glycosylation was distinct from that of UC- and SC-uEVs (**Figure 5**). The most prominent THP-associated signals suggested the presence of sialylated *N*-glycans through highest binding of *N*-acetylglucosamine- (GlcNAc) specific lectins DSA, STA, LEL and WGA (which also binds sialylated structures through a charge-mediated binding site). SNA-I, which recognizes *N*-acetylneuraminic acid- (NeuAc-) α-(2,6)-Gal/GalNac epitopes showed intermediate binding intensity as did lectins which recognize *N*-acetyllactosamine (LacNAc) found in complex *N*-linked oligosaccharides. AAL, a lectin reported to recognize fucose- (Fuc-) α-(1,6)- modifications which are common components of mammalian *N*-glycans [51], also demonstrated readily detectable binding to all THP samples.

Although multiple THP-binding lectins also bound to uEVs, the vesicle preparations showed a substantially greater variety of lectin interactions with less pronounced dynamic range between the lowest and highest intensity events. Notably, uEV samples isolated from three healthy adults demonstrated little inter-individual variation. AIA did not bind to THP, but positive binding to uEVs suggested the presence of β-Gal terminations. Several additional Gal/GalNAc-specific lectins as well as mannose binding lectins, GNA and HHA, also bound with low and intermediate intensities to uEV samples but not THP. Deviations in responses across technical replicates for uEVs and THP were modest at most lectins (**Figure 5**).

Unsupervised hierarchical clustering of all replicate profile data produced clear separations between THP, SC- and UC-uEV samples (**Figure 5**). The separation between THP and UC-uEVs was greater than that between THP and SC-uEVs (similarity division point 0.28 vs 0.57, **Figure 5**). While this might be indicative of an influence of co-purified THP on the profile of SC-uEVs, this factor alone is unlikely given that THP was also a significant component in UC-uEV samples (**Figure 1B**).

### Verification of Carbohydrate-mediated Lectin Binding of uEVs

**Figure 6:**
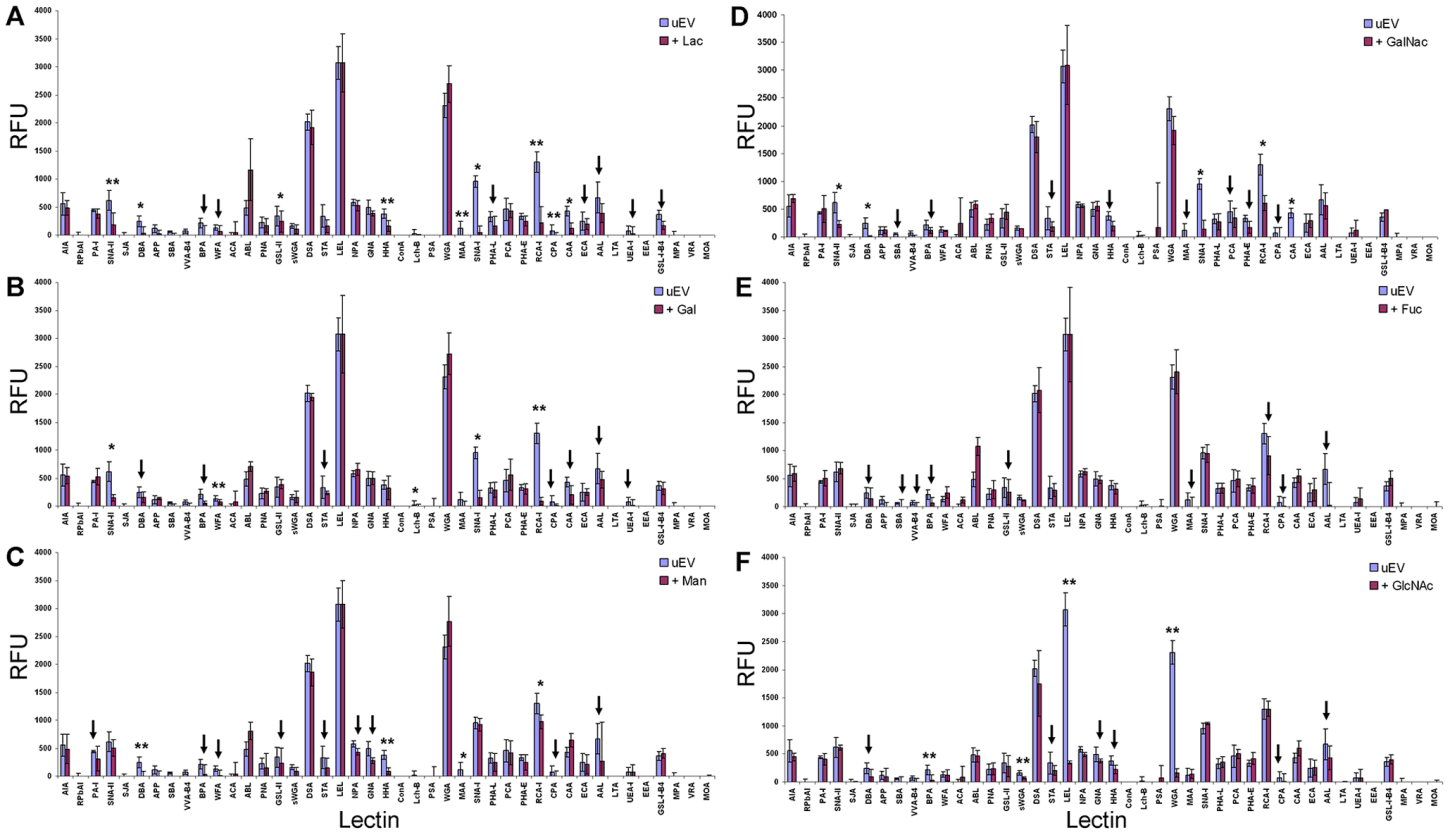
Competitive inhibition of uEV interactions with lectin microarray using six different sugars. Data normalized to the response of LEL to show the relative inhibition through competition with 50(**A**) Lac, (**B**) Gal, (**C**) Man, (**D**) GalNAc, and (**E**) and Fuc (**F**). To evaluate 50 mM GlcNAc inhibition (**F**), data was normalized to the response of RCA-I. Mean data is representative of inhibition for a single biological sample experiment conducted with 3 technical replicates. Error bars represent ± average deviation. Significance (** = *p*≤0.01, * = *p*≤0.05) determined by two-tailed, two-sampled unequal variance Student’s t-test. Arrows mark reductions in intensity greater than 20% with *p*>0.05.

**Figure 7:**
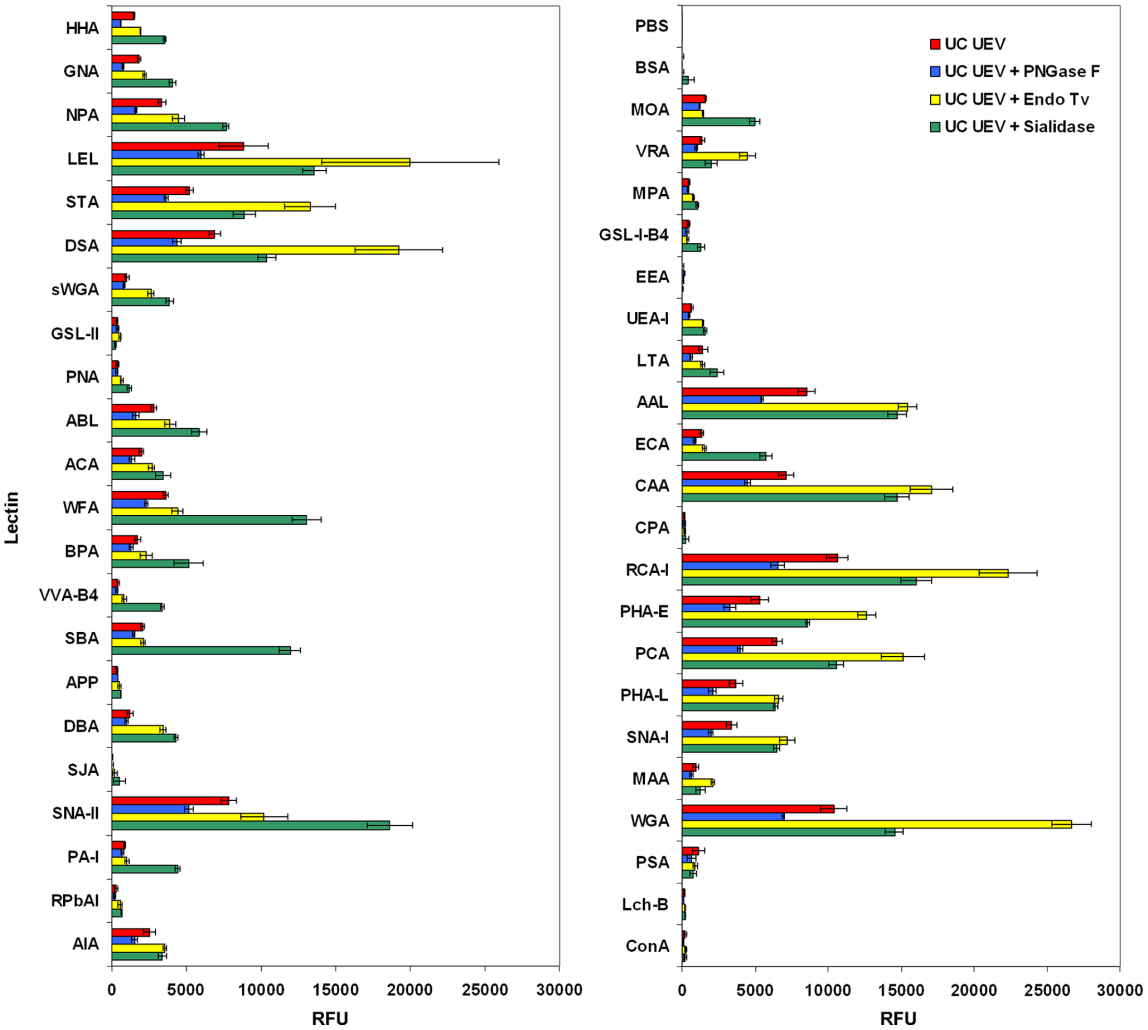
Graphical representation of results of lectin microarrays of intact urine uEVs after glycosidase and amidase treatment. Combined panels represent the complete set of microarray features containing 43 lectins and two negative controls (PBS and BSA). All data shown represent the mean ± average deviation of 3 technical replicates carried out on a single biological sample.

To confirm that lectin microarray binding of labeled uEVs was carbohydrate-mediated, two approaches were taken. First, UC-uEVs were incubated with lectin microarrays in the presence of competitive sugar inhibitors. In these analyses, the majority of lectins showed expected reductions in binding intensity in the presence of haptenic inhibitors (**Figure 6A through F**). Next, aliquots of a single UC-uEV preparation were subjected to hydrolysis with carbohydrate-specific enzymes prior to lectin microarray analysis. As shown in **Figure 7**, the amidase PNGase F, which cleaves most *N*-glycans from glycoproteins, produced uniformly-reduced intensities across many lectins. Endo Tv, a high-mannose-specific *endo*-β-*N*-glycosidase [52], produced a substantial increase in intensity at a variety of lectins characterized as having affinity for more complex structures including those with extended biantennary and bisecting GlcNAc motifs (e.g. PHA-E). This observation was consistent with an ‘unmasking’ effect resulting from the selective removal of high-mannose *N*-glycans by Endo Tv hydrolysis. Neuraminidase treatment produced predicted increases in many Gal/GalNAc-specific lectin responses. Taken together, the resulting changes in lectin profiles obtained through haptenic inhibition and enzymatic modification verified that the lectin microarray profiles were the result of carbohydrate-specific interactions.

### Pilot Screening of uEVs from ADPKD Patients and Matched Controls

**Figure 8:**
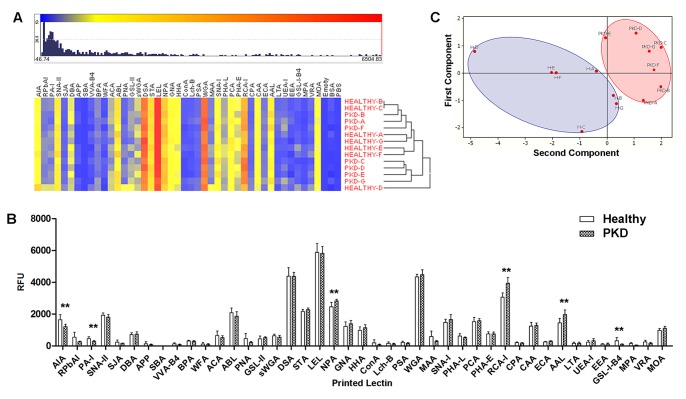
Comparison of ADPKD uEV profiles and matched controls. (**A**) Heat map and distribution of intensities for clustered ADPKD gUC-uEV (PKD) mean normalized lectin array responses and age-matched gUC-uEV controls (HEALTHY). Mean data representative of 3 (H-A, H-B, H-D, H-E, H-F, H-G) or 2 (H-C) technical replicates for the indicated 7 healthy uEV samples and of 4 technical replicates for the 7 ADPKD (PKD-A through PKD-G) uEV samples. (**B**) Comparison of pooled responses (all PKD data combined vs all HEALTHY combined). Error bars represent ± average deviation. Significance (** = *p*≤0.01) determined by paired, two-tailed Student’s t-tests of individual lectin responses. (**C**) Score plot generated from data of the 6 significant lectin responses indicated in (B) showing grouped ADPKD (PKD, red oval) and matched healthy (H, blue oval) responses.

Having established lectin microarray profiling of uEVs from urine of healthy volunteers, a pilot study was performed on uEVs prepared by density gradient ultracentrifugation (gUC) from seven adult subjects with ADPKD with preserved renal function and from seven age-matched healthy subjects (**Table 1**). Heat-mapped responses of mean (3 arrays per donor) profiles for all 14 subjects after unsupervised clustering are shown in **Figure 8A**. Although one sample demonstrated outlier behavior (sample HEALTHY-D, **Figure 8A**), the profiles across samples were closely comparable. No overall separation of ADPKD and healthy control profiles was demonstrated by hierarchical clustering, suggesting that ADPKD was not associated with a broad alteration to the surface glycosylation profile of uEVs. However, comparative analysis of the mean intensities for individual lectins showed modest, but significant, differences between ADPKD and healthy controls for lectins AIA, PA-I, NPA, RCA-I, AAL, and GS-I-B4 (**Figure 8B**). Principal component analysis performed using only the combined data for the six lectins which demonstrated significant differences resulted in distinct groupings for ADPKD and healthy controls (**Figure 8C**). This pilot study illustrated the potential for utilizing lectin microarray to broadly profile uEV surface glycosylation during health and disease and as a prelude to identifying carbohydrate-based biomarkers of kidney disease.

**Table 1:** Clinical details of ADPKD patients and matched healthy subjects.

Subject	Gender	Age	eGFR
ADPKD_A	Male	37	>60
ADPKD_B	Female	33	>60
ADPKD_C	Male	35	>60
ADPKD_D	Male	29	>60
ADPKD_E	Female	34	>60
ADPKD_F	Female	22	>60
ADPKD_G	Male	33	>60
Healthy_A	Male	34	>60
Healthy_B	Male	33	>60
Healthy_C	Male	35	>60
Healthy_D	Female	36	>60
Healthy_E	Female	37	>60
Healthy_F	Female	24	>60
Healthy_G	Male	25	>60

## Discussion

To date, there has been relatively little analysis of uEV glycosylation in comparison to the profiling of uEV-associated proteins and RNA. This study further develops the lectin-based FCM and lectin microarray approaches as sensitive platforms capable of detecting differential glycosylation on intact uEVs. Profiles generated for a pilot cohort of ADPKD patient and matched control samples highlight the potential of the lectin microarray method and suggest that even minor differences in glycosylation may be primarily identified by this approach.

Diverse lectin binding profiles of uEVs hint at the broad variety of oligosaccharides presented on their surfaces. Our lectin profile data compares favorably to the limited amount of data available from recent studies of uEV glycosylation. For example, the mass-spectrometric identification of released *N*-glycans used by Staubach *et al*. to identify high mannose forms as comprising between 73 and 81% of the population and 18 to 26% as the sialylated, complex type (both biantennary and triantennary forms) from healthy uEVs [28] is in agreement with our observation of uEV sensitivity to Endo Tv and PNGase F treatment. While enzymatic removal of many classes of *N*-glycans was evident from reduced signals across the lectin microarray, the binding of lectins which typically recognize mucin-type *O*-linked motifs, such as Tn- or T-antigen, did not show increased intensity as might have been expected after removal of the presumably larger *N*-glycans occupying the surface of the vesicles. This may indicate low abundance of mucin-type *O*-linked glycans, a greater abundance of more extended *O*-linked structures, or continued steric interference of additional surface structures. While these *in vitro* enzyme-treated uEV responses cannot verify the structures involved, the modulation is a demonstration of the potential of lectin microarray screening to detect similar changes in uEV surface glycosylation profiles as a consequence of disease.

Recently, Batista *et al* reported that EVs isolated from four cell lines and human breast milk demonstrated a common interaction with LEL and DSA [29], two lectins which also showed prominent and inhibitable signals with uEVs on our lectin microarrays. Conservation of EV glycosylation from different cell types may suggest that a specific subset of glycosylation events is associated with EV formation. The limited inter-individual variation observed amongst healthy donor uEV glycosylation profiles also supports this concept. Recent proteomic studies indicate that the number of glycoproteins contributing to the lectin profiles we observed is likely to be high [7], [16], [19], [53]. Variations of the glycosylation of individual proteins has been associated with kidney diseases and metabolic disorders which affect kidney function such as those in hinge-region, *O*-linked glycosylation associated with IgA-mediated nephropathies [54], [55] and podocyte flattening suggested to be dependent upon incorrect glycosylation of alpha-dystroglycan [56]. Whether specific glycoproteins that are enriched in uEVs manifest altered oligosaccharide composition during kidney disease will require further investigation. Furthermore, it should be noted that lectin profiles of uEVs may also be influenced by the presence of carbohydrates conjugated to membrane lipid structures (glycolipids) [57]. Thus, it is likely that glycolipids make an important contribution to uEV glycoprofiles and that these also become altered in response to kidney disease. Full characterization of uEV glycosylation from specific patient groups will require the combined application of affinity-based approaches and high-performance structural carbohydrate analysis techniques such as liquid chromatography and mass spectrometry [31].

The co-purification of THP, a heavily glycosylated protein that is present in large amounts in urine and readily precipitates during sample handling, is a potential challenge to analysis and interpretation of affinity-based uEV glycosylation profiles. Previous reports indicate that THP is extensively modified with *N*-linked glycans and may also have *O*-linked structures during pregnancy [58]–[60]. A variety of strategies to reduce the inclusion of THP in uEV samples have been reported although purification of adequate quantities of THP-free uEVs remains problematic [41], [47], [48]. For this study, strict adherence to specific temperature and handling criteria was applied to minimize THP inclusion and we observed that the addition of reducing agents during uEV isolation [41] was only slightly more effective and did not alter the result of subsequent FCM and lectin microarray profiling (data not shown). Importantly, while THP was detected to a variable degree in all uEV samples regardless of the method (SC, UC or gUC) used for enrichment, our comparisons of uEV preparations with purified THP in FCM and lectin microarray assays, essentially rule out the possibility that co-purified THP was responsible for a large component of the uEV profiles that were observed.

In conclusion, our results provide the first comprehensive profile of the surface glycosylation characteristics of uEVs of healthy adults prepared by both ultracentrifugation- and concentration-based methods. The complexity of this profile, as reflected in the results of FCM and lectin microarray assays, indicates that there is much to be learned from further exploration of the finer details of uEV protein and lipid glycosylation. Our results from a pilot study of ADPKD samples suggest that glyco-analysis of uEVs from this and other forms of kidney disease can serve as a platform for biomarker identification.

## Materials and Methods

### Ethics Statement

The collection of urine samples from healthy adult volunteers for preparation of extracellular vesicles for this study was specifically approved by the Galway University Hospital Ethics Committee and was carried out following written informed consent. The collection of urine samples from adult patients with ADPKD and from age-matched healthy adults for preparation of extracellular vesicles for this study was specifically approved by the Mayo Clinic Institutional Review Board and was carried out following written informed consent.

### Enrichment and Labeling of Urinary uEVs

First void, clean-catch urine samples were collected from healthy adult volunteers in sterile receptacles containing EDTA-free broad-spectrum protease inhibitor (Roche Diagnostics, West Sussex, UK). Urine samples were held at room temperature (RT) for no more than 2 hours from collection through clarification. Urine was clarified by centrifugation at 1800×*g* for 10 min. Supernatants were frozen at −80°C in 25 mL aliquots until use. First void urine used to prepare gUC-uEVs was processed immediately without freezing.

Prior to ultracentrifugation (UC) and centrifugal concentration (spin concentration, SC), frozen samples were thawed on ice, heated to 40°C for 30 min to allow complexes of proteins to dissociate completely, and then vortexed continuously for 2 min. Aliquots were transferred to 45 mL high-speed centrifuge tubes (Nalgene, Thermo-Fisher, Dublin, Ireland) and centrifuged at 17,000×*g*, 20°C, for 15 min.

SC enrichment of uEVs using MWCO centrifugal filtration devices was performed in a similar manner to previously reported methods [41], [47] with the exceptions that the entire process was carried out at 20°C and without the use of a reducing agent. Briefly, 15 mL of 17,000×*g* supernatant was applied to a 100 kDa MWCO centrifugal filtration device (Millipore, Billerica, MA, USA) and centrifuged at 1500×*g*, 20°C until the total volume was reduced to approximately 200 µL. The remaining supernatant was applied to the spin device, and centrifugation continued until a final volume of 200 µL was reached. Concentrated uEVs were pipetted from the spin device and the device was washed with 100 µL of 20 mM phosphate buffered saline, pH 7.4, (PBS) and the two volumes combined.

Ultracentrifugation (UC) enrichment of uEVs was performed according to published protocols [29], [41]–[45], [48]. 17,000×*g* supernatant was transferred to 36 mL Polyallomer tubes (Kendro, Ashville, USA) for centrifugation at 110,000×*g*, 17°C, for 2.5 h in a Sorvall 100SE Ultra Centrifuge (Thermo-Fisher). The clear-white, breakable uEV pellet was recovered in PBS and stored at 4°C overnight. The pellet suspension was further concentrated in a 0.5 mL, 100 kDa centrifugal filter unit (Millipore) and frozen at -80°C until use.

Density gradient ultracentrifugation (gUC) uEV enrichment was performed according to the previously reported method [19] with minor modifications. Briefly, 270 mL of fresh, first void urine was collected from each individual and briefly centrifuged at 4000×*g* to remove cells and debris and then centrifuged at 150,000×*g* for 1 h to pellet a mixture of THP and uEVs (crude uEVs). THP was removed from the crude uEVs by centrifugation on a 5–30% sucrose gradient in D_2_O (200,000×*g* for 24 h). Three distinct bands of uEVs were visible by light scattering when the sucrose D_2_O gradient was illuminated along its long axis. The three bands were designated as A, B and C; A being the lightest (RI- η = 1.3436 sd+/−0.00124), B the band of intermediate density band (ADPKD-uEVs, (RI η = 1.3539 sd+/−0.000831) and C the highest density (RI η = 1.3625 sd+/−0.000911). ADPKD-uEVs (Band B) were recovered using a BioComp fractionation device (BioComp Instruments, Inc., Fredericton, Canada) and diluted 4-fold in PBS in normal water with 1×EDTA-free broad-spectrum protease inhibitor (Roche). 1 µL of 5 mg/mL SP-DilC18(3) (Invitrogen, Life Technologies, Carlsbad, CA, USA) was added to label lipid vesicles, and the mixture centrifuged in a Sorvall Surespin 630 rotor (Thermo-Fisher) at 120,000×*g* overnight to recover uEVs, which appeared as a pink dot on the bottom of the tube. This was resuspended in 200 µL of 0.25 M sucrose in 20 mM MES, pH 6.0, with 1×EDTA-free broad-spectrum protease inhibitor (Roche). Finally, the uEV concentration was estimated using bicinchonic acid method (BCA, Thermo-Fisher) and uEVs were frozen at −80°C.

Urinary extracellular vesicles prepared by UC and SC methods were fluorescently labeled with the lipophillic dye PKH26 (Sigma Aldrich, St. Louis, MO) following the manufacturers’ protocols. All steps were carried out at room temperature and in the dark. Excess dye was removed from labeled uEVs by filtration. Briefly, a 4 mL, 100 kDa MWCO spin device (Millipore) was pre-washed by centrifugation with PBS and the entire final volume of the uEV labeling mixture added. An additional 2 mL PBS was added prior to centrifugation for 20 min, 2000×*g*, at room temperature. An additional 1 mL of PBS was added to the spin device and a final centrifugation at 2000×*g* was used to reduce the final volume to approximately 50 µL. The retentate was removed and the bottom of the spin device was rinsed with an additional 20 µL of PBS. Labeled uEVs were frozen at −80°C until use.

### uEV Size Determination

Dilute samples of uEVs from a single healthy donor enriched by SC and UC methods were subjected to particle size determination by the IZON qNano (Izon Science, Ltd., Christchurch, NZ) particle analysis system [49].

### Transmission Electron Microscopy

Sample preparation was carried out using the droplet method as previously described [44]. Briefly, 25 µL (4.65 µg) of uEVs in PBS were fixed with 4% paraformaldehyde in a 1∶1 ratio for 20 min on a 200 mesh, formvar-coated, copper grid (Agar Scientific, Ltd., Essex, UK) then washed twice in 100 µL PBS for 1 min followed by cross-linking with 50 µL 1% glutaraldehyde for 5 min. The sample was washed three times in 100 µL PBS for 1 min. Contrast staining was performed with 50 µL 2% uranyl acetate: 2% methylcellulose (1∶10) for 10 min on ice. Imaging was done with a Hitachi H7500 TEM (Hitachi HTI, Leixlip, Ireland) at 75 kV.

### Isolation and Labeling of Tamm-Horsfall Protein

THP was isolated using a modification of the original method [59]. 50 mL aliquots of urine from healthy donors were clarified by centrifugation at 400×*g*, 20°C, for 10 min. To the resulting supernatants, 1.7 g NaCl was added, the mixture vortexed briefly and placed on ice for 2 h after which the resulting precipitate was recovered by centrifugation at 1800×*g* for 15 min at 4°C. The pellet was resuspended in room temperature, 15 MΩ water (dH_2_O), vortexed extensively and 1.7 g NaCl added before returning the mixture to ice for an additional 2 h. This process was repeated 3 times. After the final precipitation and centrifugation, the pellet was resuspended in 10 mL dH_2_O and dialyzed twice against 2 L dH_2_O for 4 h at RT using a 15 kDa MWCO membrane (Spectrum Laboratories, Rancho Dominguez, CA, USA). Following dialysis, THP was completely lyophilized and stored at 4°C until use. Covalent labeling of THP with AlexaFluor® 647 was performed using the manufacturer’s protocol (Life Technologies). Excess dye was separated from labeled THP by exhaustive dialysis against dH_2_O.

### SDS-PAGE and Immunoblotting

uEV, HSA and THP samples were mixed with loading buffer containing 25% β-mercaptoethanol, denatured at 100°C for 5 min and separated in NuPAGE 4–12% Bis-Tris gels using MOPS running buffer (both Life Technologies) at 150 V constant for approximately 1 h. Gels were either stained with 0.05% (w/v) Coomassie G-250 in a fixative solution containing 30% ethanol and 10% acetic acid then partially de-stained with distilled de-ionized water (ddH_2_O) or stained with silver (Pierce, Thermo-Fisher). Gels were imaged using a ChemiDoc® imaging system (Bio-Rad, Hercules, CA, USA). Alternatively, unstained proteins (5 µg uEV for CD24, 4 µg uEV for AQP2) were transferred to 0.22 µm polyvinylidene fluoride membrane (Millipore), washed in methanol and rinsed in transfer buffer. Two distinct transfer techniques were used with uEVs; for AQP2 probing, the semi-dry transfer apparatus (Bio-Rad) was employed and for CD24, a wet transfer (Bio-Rad) was used. Semi-dry transfer was performed at 1.5 mA/cm^2^ for 30 min; wet transfer was performed at 100 V for 90 min. Membranes were blocked directly after transfer with 1.5% non-fat dry milk in TBST overnight at 4°C. Following washing, membranes were incubated with 1∶10 dilution of hybridoma medium containing anti-CD24 antibody (Prof. Peter Altevogt, Heidelberg, Germany) diluted in TBST. Polyclonal anti-AQP2 antibody (rabbit, Sigma Aldrich, A7310) was used at 1∶1000 dilution in TBST supplemented with 5% BSA for 4 h at 4°C. Blots were incubated with secondary antibodies against mouse or rabbit conjugated to HRP diluted 1∶5,000 in TBST for 1 h at RT. Detection was performed by enhanced chemiluminescence (Thermo-Fisher) with an Image Station 4000 MM Pro® (Carestream, Woodbridge, CT, USA).

### Flow Cytometric Analysis of uEVs and THP

All processes were carried out at room temperature unless otherwise specified. uEVs or THP were adsorbed onto aldehyde-sulfate functionalized latex beads according to a published protocol [46]. 5 µg of uEVs or THP in 40 µL of PBS were absorbed to 10 µL of 4 µm latex beads (4% (w/v) (Invitrogen, cat no. A37304, Life Technologies) for 15 min. 950 µL of PBS was then added and the conjugation reaction allowed to proceed overnight at 4°C. Beads were blocked with 110 µL of 1 M glycine solution in PBS for 30 min. The beads were centrifuged for 3 min at 1700×*g* and the supernatant was removed and replaced with 1 mL of PBS with 1% bovine serum albumin (PBS-B) and this step was repeated 3 times. Finally, beads were re-suspended in 0.5 mL of PBS-B.

For incubation with affinity molecules, 50 µL aliquots of PBS containing an optimized concentration of each biotinylated lectin was mixed with 10 µL of re-suspended beads in the wells a 96 well plate and were incubated for 30 minutes at 4°C followed by 3 steps of washing with 200 µL of PBS-B. Lectins were used at the following concentrations: PNA, PHA-E, RCA-I (Vector Laboratories, Peterborough, UK) at 0.5 µg per 50 µL in PBS-B, SNA-I, MAA, WFA (EY Laboratories, San Mateo, CA, USA) 0.75 µg per 50 µL and GSL-I-B4, AAA, Jacalin (AIA) (EY Laboratories) at 1 µg per 50 µL in PBS-B.

For secondary incubation, 10 µL of a 1∶40 dilution of streptavidin-allophycocyanin (SA-APC, BD Biosciences, Oxford, UK) in PBS-B was added and incubated in the dark for 30 minutes at 4°C followed. Alternatively, the beads were incubated with 50 µL of a 1∶10 dilution of anti-CD24 hybridoma medium in PBS followed by anti-mouse IgG-APC (BD Biosciences). The stained beads were washed 3 times in PBS-B then re-suspended in 300 µL of PBS-B and analyzed with a FACSCanto® flow cytometer (BD Biosciences) and Flowjo® version 7.6.4 (TreeStar, Inc., Ashland, OR, USA) software. For all conditions, 5000 events were recorded for each of 5 replicates. Median fluorescence intensities (MFI) for APC were recorded for each experimental and control sample and the binding value for each lectin was calculated as fold-increase over control according to the following formula:

Fold Increase = [(Average MFI of uEV-beads+lectin/SA-APC)/(Average MFI of control-beads+lectin/SA-SPC)].

### Enzymatic Treatment of uEVs and THP

Parent stocks of labeled uEVs were prepared as described above. In PCR tubes, 2 µL (approximately 1 µg) of intact uEVs was added to 2 µL of 50 mM sodium phosphate buffer, pH 5.0, for glycosidase treatment or pH 7.0 for amidase treatment. 1 mU of each respective enzyme was added to the buffered uEVs and incubated in the dark at 37°C for 3 hours. Enzymes used: PNGase F (Calbiochem, Merck), Sialidase from *Vibrio cholerae* (Calbiochem, Merck), β-galactosidase from bovine testes (Sigma Aldrich), and endo-glycosidase Tv (Endo Tv) from *Trichoderma viride* (prepared in-house, [52]). Enzyme-treated samples were diluted in TBST as below prior to lectin microarray incubation.

### Lectin Microarray Preparation

Forty three lectins sourced from multiple vendors were diluted to 0.5 mg/mL in PBS supplemented with 1 mM of respective haptenic sugar to maintain binding site integrity (see **Table S1 in Supporting Information File S1**). Lectins were printed on Nexterion® H (Schott, Mainz, Germany) functionalized glass slides using a Scienion S3 non-contact spotter (Scienion, Berlin, Germany) under constant 62% (+/−2%) relative humidity at 20°C. Following printing, slides were incubated in a humidity chamber overnight to ensure completion of covalent conjugation. Unoccupied functional groups were deactivated by incubation with 100 mM ethanolamine in 50 mM sodium borate, pH 8. The slides were washed with PBS with 0.05% Tween-20 (PBS-T) three times and once with PBS, centrifuged dry (450×*g*, 5 min) and stored dry at 4°C with desiccant until use.

### Optimization of Lectin Microarrays for Profiling uEVs and THP

Lectin microarray incubation was carried out as previously described [61]. All processes were carried out with attention to limit light exposure and all experiments included a control glycoconjugate to monitor performance of the arrays and imaging equipment. Microarray slides were incubated using an eight-well gasket slide and incubation cassette system (Agilent Technologies, Cork, Ireland). 70 µL of each sample in TBST with 1 mM CaCl_2_, 1 mM MgCl_2_ was applied to each well of the gasket. The microarray slide was sandwiched with the gasket, the cassette assembled and placed in a rotating incubation oven (23°C, approximately 4 rpm) for 40 min. Slides were disassembled under TBST, washed once in TBST for 2 min in a Coplin jar and rinsed with TBS. The microarrays were dried by centrifugation (450×*g* for 5 min) and scanned immediately using either a Perkin-Elmer Scanarray HT (543 nm or 633 nm channel, 90% laser power, 70% PMT, Perkin-Elmer, Waltham, MA, USA) or Agilent G2505 (532 nm or 633 nm channel, full power, 90% PMT, Agilent) microarray scanner.

### Haptenic Sugar Inhibition of uEV Binding

uEVs were diluted in respective 50 mM solutions of galactose (Gal), lactose (Lac), *N*-acetylgalactosamine (GalNAc), *N*-acetylglucosamine (GlcNAc), mannose (Man), or fucose (Fuc) (all from Sigma-Aldrich) in TBST and incubated in parallel subarrays on the same slide as the uninhibited sample.

### Microarray Data Extraction and Analysis

Raw intensity values were extracted from the image files using GenePix Pro v6.1.0.4 (Molecular Devices, Sunnyvale, CA, USA) and a proprietary *.gal file using adaptive diameter (70–130%) circular alignment based on 230 µm features and exported as text to Excel (Microsoft, WA, USA). Local background-corrected median feature intensity data (F543median-B543) was selected for analysis. The median of six replicate spots per subarray was handled as a single data point for graphical and statistical analysis.

For healthy and ADPKD uEV comparisons as well as THP, lectin microarray intensity values were normalized to the median total intensity value for all features across all subarrays in a single experiment (preliminary experiments with healthy uEVs D1, D2 and D3, n = 18; THP D1, D2 and D4, n = 13; ADPKD patients A through G, n = 28; matched healthy controls A through G, n = 20). For inhibition experiments carried out on PKH26 labeled ELVs, individual profiles were adjusted to an uninhibited lectin. For Gal, GalNAc, Lac, Man, and Fuc inhibitions, lectin LEL was chosen, for GlcNac inhibition, RCA-I. The significance of inhibition data was evaluated using a standard Student’s t-test (two-tailed, two-sample unequal variance). For enzymatically-treated uEVs, only equal sample normalization was employed and the data presented in histogram form of average intensity with standard deviation of three experimental replicates of the median of six feature replicates for one experiment (18 data points).

Unsupervised, hierarchical clustering of uEV and THP lectin binding data was performed with Hierarchical Clustering Explorer v3.0 (http://www.cs.umd.edu/hcil/hce/hce3.html). Previously normalized data for uEVs was clustered with the following parameters: No pre-filtering, complete linkage, Euclidean distance. Combined THP and uEV lectin microarray data were normalized by rescaling range to 0 to 65200, approximately equivalent to the dynamic range of the imaging software, prior to clustering to remove bias. For pooled ADPKD and control microarray data, statistical significance of differences between lectin microarray responses was established by paired, two-tailed Student’s t-tests for each individual lectin. Principal component analysis of previously normalized data was performed using Minitab® 16 (version 16.1.1, Minitab, Inc., State College, PA, USA). Microarray data in MS-DOS, tab-delimited text format are freely available from the authors to interested researchers.

## Supporting Information

File S1 Table S1. Lectin microarray composition list; Figure S1. Result of IZON® particle size distribution analysis for both UC (pink) and SC (blue) uEV preparations from a single healthy subject. The particle size mode and range for both samples was determined to be approximately the same; Figure S2. Flow cytometric analysis of uEVs and THP. Typical responses for antibody- or lectin-stained (a) uEVs and (b) THP. These responses were generated using 5 µg unlabeled uEVs or 20 µg unlabeled THP.
(DOC)
