# Supplementary material for: Surface Glycosylation Profiles of Urine Extracellular Vesicles
Source: PLoS One. 2013 Sep 19;8(9):e74801. doi: 10.1371/journal.pone.0074801 (PMC3777961; doi:10.1371/journal.pone.0074801)
Supplement: File S1 — Table S1. Lectin microarray composition list; Figure S1. Result of IZON® particle size distribution analysis for both UC (pink) and SC (blue) uEV preparations from a single healthy subject. The particle size mode and range for both samples was determined to be approximately the same; Figure S2. Flow cytometric analysis of uEVs and THP. Typical responses for antibody- or lectin-stained (a) uEVs and (b) THP. These responses were generated using 5 µg unlabeled uEVs or 20 µg unlabeled THP. (DOC) [file pone.0074801.s001.doc]

**Supporting Information File S1**

**Surface Glycosylation Profiles of Urine Extracellular Vesicles**

Jared Q. Gerlach PhD^1, 2^, Anja Krüger MSc^1, 2^, Susan Gallogly, MSc^1^; Shirley A. Hanley PhD^1^, Marie C. Hogan MD, PhD^3^, Christopher J. Ward MB ChB, PhD^3^, Lokesh Joshi PhD^2,†^, and Matthew D. Griffin MD^1,†^

^1^Regenerative Medicine Institute (REMEDI), National Centre for Biomedical Engineering Science, National University of Ireland, Galway, Ireland.

^2^Glycoscience Group, National Centre for Biomedical Engineering Science, National University of Ireland, Galway, Ireland.

^3^Dept. of Medicine, Division of Nephrology and Hypertension, Mayo Clinic, Rochester, MN, USA.

^†^ These authors contributed equally as senior authors.

**Table S1. Lectin microarray composition list.**

| **Lectin Number** | **Plate Position** | **Abbreviation** | **Origin** | **Species** | **Common name** | **Major Ligand(s)** | **Vendor** |
| --- | --- | --- | --- | --- | --- | --- | --- |
| 1 | A01 | AIA, Jacalin | Plant | *Artocarpus integrifolia* | Jack fruit lectin | Gal (sialylation independent) | EY Labs |
| 2 | A02 | RPbAI | Plant | *Robinia pseudoacacia* | Black locust lectin | Gal, GalNAc | EY Labs |
| 3 | A03 | PA-I | Bacteria | *Pseudomonas aeruginosa* | Pseudomonas lectin | Gal, Gal derivatives | Sigma Aldrich |
| 4 | A04 | SNA-II | Plant | *Sambucus nigra* | Sambucus lectin-II | Gal/GalNAc | EY Labs |
| 5 | A05 | SJA | Plant | *Sophora japonica* | Pagoda tree lectin | βGalNAc | EY Labs |
| 6 | A06 | DBA | Plant | *Dolichos biflorus* | Horse gram lectin | GalNAc | EY Labs |
| 7 | A07 | APP | Plant | *Aegopodium podagraria* | Ground elder lectin | GalNAc | EY Labs |
| 8 | A08 | SBA | Plant | *Glycine max* | Soy bean lectin | GalNAc | EY Labs |
| 9 | A09 | VVA-B4 | Plant | *Vicia villosa* | Hairy vetch lectin | GalNAc | EY Labs |
| 10 | A10 | BPA | Plant | *Bauhinia purpurea* | Camels foot tree lectin | GalNAc/Gal | EY Labs |
| 11 | A11 | WFA | Plant | *Wisteria floribunda* | Japanese wisteria lectin | GalNAc/Sulfated GalNAc | EY Labs |
| 12 | A12 | ACA | Plant | *Amaranthus caudatus* | Amaranthin | Sialylated/Gal-β(1,3)-GalNAc | Vector Labs |
| 13 | A13 | ABL | Fungi | *Agaricus bisporus* | Edible mushroom lectin | Gal-β(1,3)-GalNAc, GlcNAc | EY Labs |
| 14 | A14 | PNA | Plant | *Arachis hypogaea* | Peanut lectin | Gal-β(1,3)-GalNAc | EY Labs |
| 15 | A15 | GSL-II | Plant | *Griffonia simplicifolia (Bandeiraea simplicifolia)* | Griffonia/Bandeiraea lectin-II | GlcNAc | EY Labs |
| 16 | A16 | sWGA | Plant | *Triticum vulgaris* | Succinyl WGA | GlcNAc | EY Labs |
| 17 | A17 | DSA | Plant | *Datura stramonium* | Jimson weed lectin | GlcNAc | EY Labs |
| 18 | A18 | STA | Plant | *Solanum tuberosum* | Potato lectin | GlcNAc oligomers | EY Labs |
| 19 | A19 | LEL | Plant | *Lycopersicum eculentum* | Tomato lectin | GlcNAcβ1-4GlcNAc | EY Labs |
| 20 | A20 | NPA | Plant | *Narcissus pseudonarcissus* | Daffodil lectin | Man-α(1,6)- | EY Labs |
| 21 | A21 | GNA | Plant | *Galanthus nivalis* | Snowdrop lectin | Man-α(1,3)- | EY Labs |
| 22 | A22 | HHA | Plant | *Hippeastrum hybrid* | Amaryllis agglutinin | Man-α(1,3)-Man-α(1,6)- | EY Labs |
| 23 | A23 | ConA | Plant | *Canavalia ensiformis* | Jack bean lectin | Man | EY Labs |
| 24 | A24 | Lch-B | Plant | *Lens culinaris* | Lentil isolectin B | Man, fucose dependent | EY Labs |
| 25 | B01 | PSA | Plant | *Pisum sativum* | Pea lectin | Man, fucose dependent | EY Labs |
| 26 | B02 | WGA | Plant | *Triticum vulgaris* | Wheat germ agglutinin | NeuAc/GlcNAc | EY Labs |
| 27 | B03 | MAA | Plant | *Maackia amurensis* | Maackia agglutinin | Sialic acid-α(2,3)-Gal | EY Labs |
| 28 | B04 | SNA-I | Plant | *Sambucus nigra* | Sambucus lectin-I | Sialic acid-α(2,6)-Gal/GalNAc | EY Labs |
| 29 | B05 | PHA-L | Plant | *Phaseolus vulgaris* | Kidney bean leukoagglutinin | Tri/tetraantennary βGal/Gal-β(1,4)-GlcNAc | EY Labs |
| 30 | B06 | PCA | Plant | *Phaseolus coccineus* | Scarlet runner bean lectin | GlcNAc in complex oligos | Sigma Aldrich |
| 31 | B07 | PHA-E | Plant | *Phaseolus vulgaris* | Kidney bean erythroagglutinin | Biantennary, bisecting GlcNAc,βGal/Gal-β(1,4)GlcNAc | EY Labs |
| 32 | B08 | RCA-I/120 | Plant | *Ricinus communis* | Castor bean lectin I | Gal-β-(1,4)-GlcNAc | Vector Labs |
| 33 | B09 | CPA | Plant | *Cicer arietinum* | Chickpea lectin | Complex glycopeptides | EY Labs |
| 34 | B10 | CAA | Plant | *Caragana arborescens* | Pea tree lectin | Gal-β-(1, 4)-GlcNAc | EY Labs |
| 35 | B11 | ECA | Plant | *Erythrina cristagalli* | Cock’s comb/coral tree lectin | Gal-β-(1, 4)-GlcNAc oligomers | EY Labs |
| 36 | B12 | AAL | Fungi | *Aleuria aurantia* | Orange peel fungus lectin | α-Fuc (1,6) | Vector Labs |
| 37 | B13 | LTA | Plant | *Lotus tetragonolobus* | Lotus lectin | α-Fuc (1,3) | EY Labs |
| 38 | B14 | UEA-I | Plant | *Ulex europaeus* | Gorse lectin-I | α-Fuc (1,2) | EY Labs |
| 39 | B15 | EEA | Plant | *Euonymous europaeus* | Spindle tree lectin | α-Gal | EY Labs |
| 40 | B16 | GSL-I-B4 | Plant | *Griffonia simplicifolia (Bandeiraea simplicifolia)* | Griffonia/Bandeiraea lectin-I | α-Gal | EY Labs |
| 41 | B17 | MPA | Plant | *Maclura pomifera* | Osage orange lectin | α-Gal | EY Labs |
| 42 | B18 | VRA | Plant | *Vigna radiata* | Mung bean lectin | α-Gal | EY Labs |
| 43 | B19 | MOA | Fungi | *Marasmius oreades* | Fairy ring mushroom lectin | α-Gal | EY Labs |

Figure S1

| Sample | Mean Dia (nm) | Mode Dia (nm) | d90/d10 | Concentration (particles/mL) | Particles counted | Particle rate  particles/min |
| --- | --- | --- | --- | --- | --- | --- |
| uEV (Spin Concentrator) | 119.6 | 103.4 | 1.6 | 1.8 x 10^11^ | 356 | 114.3 |
| uEV (Ultra-centrifugation) | 125.4 | 98.6 | 1.8 | 1.2 x 10^12^ | 809 | 294.1 |

**
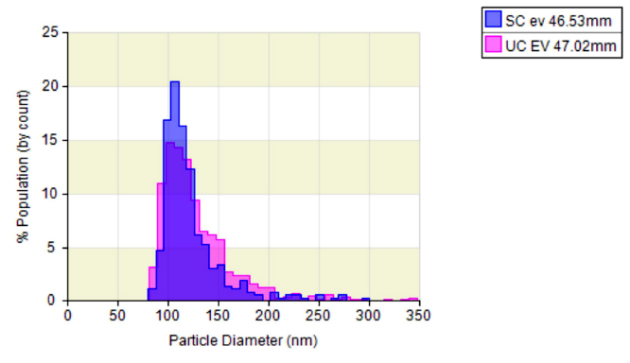
**

**Figure S1. Result of IZON® particle size distribution analysis for both UC (pink) and SC (blue) uEV preparations from a single healthy subject.** The particle size mode and range for both samples was determined to be approximately the same.

**Figure S2**


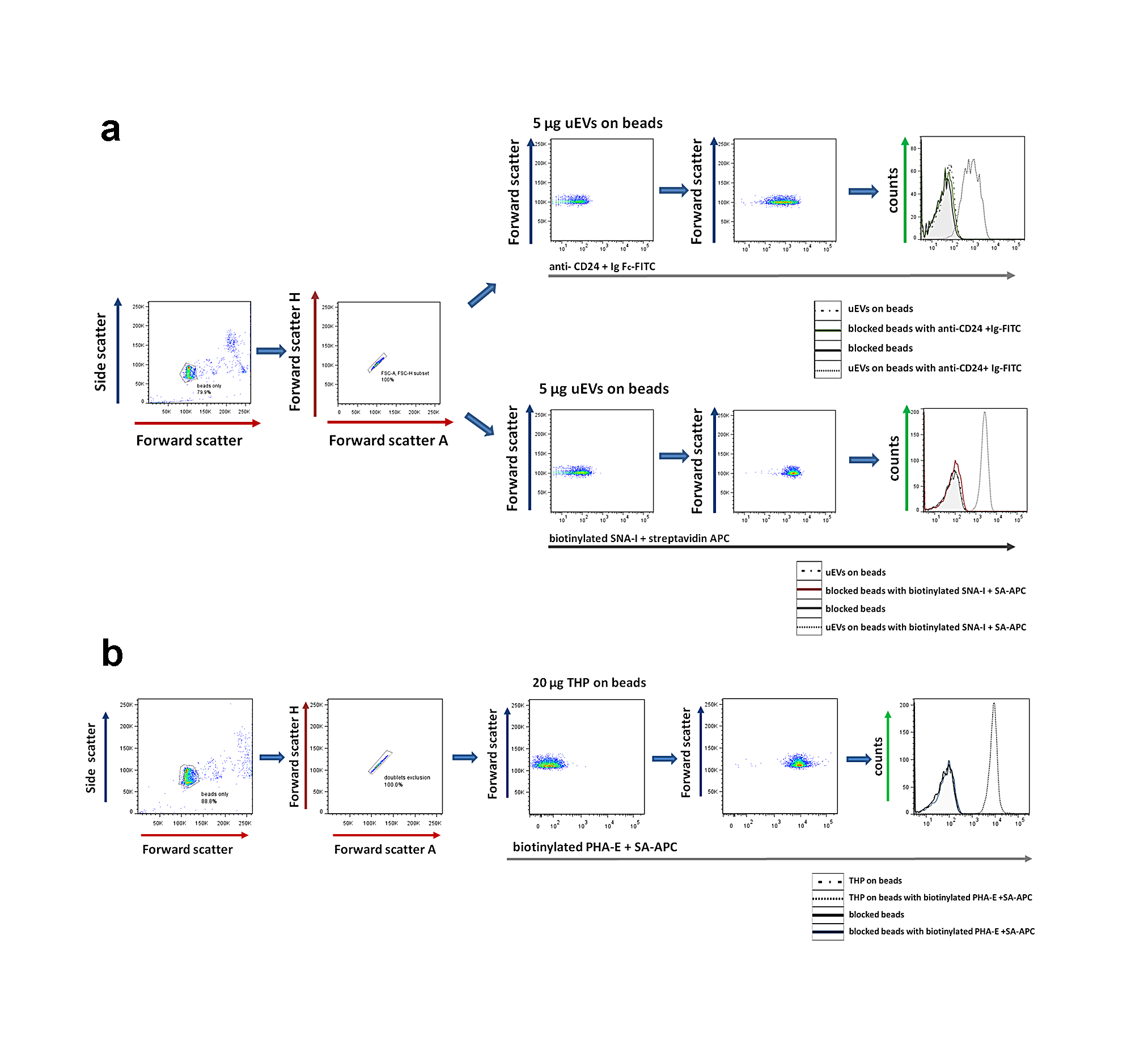


**Figure S2. Flow cytometric analysis of uEVs and THP.** Typical responses for antibody- or lectin-stained (**a**) uEVs and (**b**) THP. These responses were generated using 5 μg unlabeled uEVs or 20 μg unlabeled THP.
